# Criminal Abortion

**Published:** 1867-01

**Authors:** 


### CRIMINAL ABORTION.

We noticed a few weeks since, a small work on this subject, and made such extracts as would lead to a knowledge of the general character of the book. We then expressed the wish that every man and woman could have the facts there expressed, always before them. We find in the St. Louis Medical and Surgical Journal for December, an abstract from the report of Dr. Thaddeus A. Reamy, of Zanesville, made to the Ohio State Medical Society. We give it entire as found in the Journal:

"This subject is not introduced from choice. It is a painful duty which I can not refrain from discharging. Six years ago one of the most acute observers and one of the ablest members of this Society, then Chairman of Committee on Obstetrics, when speaking in his report of the evil, made the following prediction: "The time is not far distant when children will be sacrificed among us with as little hesitation as among the Hindoos." That prediction seemed to me at the time it was uttered, as it doubtless did to many of you, an extravagant one. But six short years have almost brought its literal fulfillment. Great God! What a state of morals for a Christian people, boasting a high degree of civilization. In my correspondence upon this subject I have found considerable reticence among medical men; many are inclined to evade a direct answer. This grows out of the delicacy of the subject, doubtless. But from a very large verbal and written correspondence in this and other States, together with personal investigation and facts accumulated, I am convinced that we have become a nation of murderers. And the victims are innocent, helpless children! Things may as well be called by their proper names. This practice is much more prevalent in the cities than in the rural districts; nevertheless, even in the country to a frightful degree. The physician is consulted as to how pregnancy can be avoided; or if it has already occurred, how can it be got rid of. With as much coolness and business-like composure, and that by the fathers or the mothers, as would characterize a consultation with a gardener as to plucking weeds from flowers. I doubt whether there are a half dozen practitioners present to-day who have not been consulted within the past year repeatedly in such cases. "We have as many children as we want," "my health is so poor I cannot bear children," etc., are phrases familiar to every physician. And be it remembered, these applicants are not confined to the poor, the indigent, or the ignorant; they are from all classes. This is one of the most alarming features of the case. The mania to destroy has gone into every avenue of society. The lettered and unlettered, rich and poor, professed christian and sinner, throng in one great mass the bloody altars, and sacrifice their offspring.

The deity who presides at such an altar ought to be carved in brass, and a copy furnished to every household which has been, or is likely to be cursed with such worship. It ought to represent a woman in the act of strangling an innocent, sweet-countenanced child; her features contorted with the heartless visage of her cruel purpose; and the second figure should represent her husband, or some lady friend, plugging her ears lest the cries of the child might move her to sympathy. Her eyes should be intently fixed upon an object in the distance, that she may not catch the countenance of the infant and behold it in the image of God, and thus be deterred from her bloody purpose. If a physician informs them when applied to, that he can not consent to such a thing, that it is murder, he will quite likely be informed that if he will not some others will; and it is to be greatly feared others do. Women learn these things from each other; many of them follow it for themselves and friends. They will use knitting needles, whalebones, a catheter, or some other instrument—rupturing the membrane. And the practice is not confined to the first three or four months of pregnancy. So intent are they, often that if success has not attended their efforts sooner, when quickening occurs, instead of having a mother's sympathy aroused, and solicitude for the welfare of her offspring, she is only reminded that she must be prompt in dispatching it before it is larger to give her trouble.

The bloody command of Pharaoh to the Hebrew midwives, that when they did the "office of midwife to the Hebrew women and saw them upon the stool, if it were a son they should kill him," has always called upon the head of that king our execrations; he has been held as a heartless monster. We have looked with horror upon the practice of the Romans in the dark ages destroying their offspring, both before and after birth, as portrayed by Juvenal in his Sixth Satire; and we have rejoiced as the mild influence of Christian civilization has controlled and enlightened the minds of the people and abolished his custom. We have wept over the heathen mother who throws her child into the Ganges as a sacrifice to her imaginary God; and we resolved to give liberally to the missionary fund that she might have the light of a better gospel. And we have made similar resolves as we read the accounts of the baby tamers in China and India. Pharaoh was a wicked and ambitious king, who had natural enmity against the Hebrews, and was jealous lest they who had been his slaves might become his conqueror. This bloody edict was therefore one of national policy. The Romans and Chinese were ignorant heathens, who knew but little of the claims of humanity, and nothing of the obligations of the Christian religion.

But what shall be said of Christian mothers murdering their offspring without cause and without conscience? And what of physicians who lend their influence and counsel in behalf of such deeds? This earth is far from being regenerated. The dark waters of the Ganges foam with the struggling of heathen infants offered in sacrifice; the bones of thousands of murdered children are bleaching at the base of the baby tamers in China. But in Christendom, yea, in our own beautiful Ohio, there prattle around the hearthstones of cottages and palaces, children who have dead infant brothers and sisters, hidden and unnamed, who were murdered by their parents' hand. What mockery for the voice of evening and morning prayer to be heard, asking God to bless and protect "Willie" and "Ellie," while from the same heart from which goes out the prayer went out the determination to destroy an unborn child! In many instances the pages of the sacred volume are turned for the reading of the morning lesson by the very hand that so recently directed the weapon for destruction. Can God hear with favor, the prayer that goes up incensed with the blood of murdered innocence? There are but few communion boards in this or perhaps any other country which have not among those who surround them at every season persons guilty of this crime.

But this subject is too revolting to contemplate. I must leave it. No doubt, some will think I have overdrawn it, or given too much time to it.

I know it is not overdrawn; and if not, too much importance can not be attached to it. It is blighting the health of thousands of women; causing ulcerations, displacements, and inflammation of the uterus. In its secondary results, it is one of the most prolific sources of moral prostitution. It is with fearful rapidity prostituting woman from the noble and God-given embodiment of virtue, innocence and affection, which has and always should make her an object of adoration and tender care, to a cruel monster whose level is far below the brute. It is filling our countries with mothers who have either committed suicide and infanticide or secured the services of a friend more skillful than themselves. I once attended a woman, wife of an intelligent, kind-hearted husband in good circumstances, mother of three lovely children, a devoted and prominent member of a Christian Church. She aborted, despite all my efforts to prevent it. She was over four months advanced. Grave symptoms supervened; purulent absorption was manifest. She died on the sixteenth day. An autopsy was had by myself and a medical friend. She had perforated the uterus very near the os with a wire which was in a male gum catheter, the wire having been thrust through the catheter in her violent efforts to destroy the fœtus. The veins throughout the whole system were filled with pus. She confessed to me, on the third day of her illness, that she had brought on the abortion in the way named. I attended her funeral; her minister preached her to the pure Paradise of God. She had committed murder and suicide. The custom is making homes that ought to echo with the matchless music of prattling children silent as the murderer's cave; the air that should be fragrant with the flowers of their planting and care, is loaded with the stench of detached and putrid ova. In short, the practice is eating as a cankering mildew at the very vitals of our holy Christianity, and unles speedily checked, will damn us as individual communities and as a nation.

What can be done? I answer, much. Medical men, from their intimate relations to the families which they attend, and from their acknowledged acquaintance with the whole subject, can command respect for their counsels in these matters, above all others. Let every honorable member of this Society, and of the entire profession, whenever consulted by a woman on this subject, candidly and patiently explain to her the nature of the crime she contemplates—set it before her in its true character.

The opinion has become prevalent among women, that if the child be destroyed before quickening, no sin attaches to the act; and that if the separation of the fœtus takes place after quickening, but before viability, though it be somewhat sinful, yet it does not amount to much. If they are counseled properly against such fallacies, they will generally desist at once. Medical men should inform them candidly and forcibly that such an act is murder at any stage of pregnancy. I am thoroughly convinced with Dr. Storer, of Boston (Prize Essay), "that the employment, in the married relation, of any means whatever, for preventing conception, is but little else than legalized prostitution. Let no physician who claims or expects to claim, respect from God or his fellow-men, be either directly or by advice, party to such an atrocity. And let every member of the profession who has already acted, or will in future act, upon these suggestions, constitute himself a vigilance committee, to bring to justice and condign punishment any and every medical man in his community who degrades himself to such an office. Let us all refuse to subscribe for and receive at our firesides, newspapers, secular or religious, in whose columns, appear advertisements of patent nostrums for "removing all irregularities, from whatever cause produced." "The proprietors would advise against women, who are in a certain condition, taking the medicine, etc., as miscarriage would occur, although, even then, there would be no danger." Much has been said, and well said, by the editor of the Medical and Surgical Reporter, Philadelphia, against the evils I have been trying to portray. This journal, an excellent one, should have our warmest commendation for its outspoken fearlessness in condemning this business. Our Western journals should also sound the alarm. Dr. Houghton, in an Obstetric report made before the Wayne County Medical Society, during the year, speaks out also in words of bold rebuke. Let every conscientious physician speak out, and every medical journal in the land, chronicle the evil. Thus can our profession come to the rescue, and signally co-operate with all other agencies of our holy Christianity in saving society from imminent impending physical, moral, and spiritual ruin.

NOTE.—Criminal prosecutions for abortions procured upon unmarried females for concealing shame, murder of the new born infant, by neglect or otherwise, for the same purposes, etc., must all cease, unless abortions among married women for convenience can be brought to an end. I can see no propriety in continuing any jurisprudence over the subject at all, unless the community is relieved of the demoralizing effects of this crime; it would soon be difficult to find a jury that would convict even in the clearest case."

